# Does online insomnia treatment reduce depressive symptoms? A randomized controlled trial in individuals with both insomnia and depressive symptoms

**DOI:** 10.1017/S0033291718001149

**Published:** 2018-05-11

**Authors:** T. van der Zweerde, A. van Straten, M. Effting, S. D. Kyle, J. Lancee

**Affiliations:** 1 Department of Clinical Psychology & EMGO Institute for Health and Care Research, VU University, Amsterdam, The Netherlands; 2 Department of Clinical Psychology, University of Amsterdam, Amsterdam, The Netherlands; 3 Sleep and Circadian Neuroscience Institute, University of Oxford, Oxford, UK

**Keywords:** CBT, depression, insomnia, online treatment, randomized controlled trial

## Abstract

**Background:**

Insomnia is effectively treated with online Cognitive Behavioral Therapy for Insomnia (CBT-I). Previous research has suggested the effects might not be limited to sleep and insomnia severity, but also apply to depressive symptoms. Results, however, are mixed.

**Methods:**

In this randomized controlled trial we investigated the effects of guided online CBT-I on depression and insomnia in people suffering from symptoms of both. Participants (*n* = 104) with clinical insomnia and at least subclinical depression levels were randomized to (1) guided online CBT-I and sleep diary monitoring (i-Sleep) or (2) control group (sleep diary monitoring only). The primary outcome was the severity of depressive symptoms (Patient Health Questionnaire-9 without sleep item; PHQ-WS). Secondary outcomes were insomnia severity, sleep diary parameters, fatigue, daytime consequences of insomnia, anxiety, and perseverative thinking.

**Results:**

At post-test, participants in the i-Sleep condition reported significantly less depressive symptoms (PHQ-WS) compared with participants in the sleep-diary condition (*d* = 0.76). Large significant effects were also observed for insomnia severity (*d* = 2.36), most sleep diary parameters, daytime consequences of insomnia, anxiety, and perseverative thinking. Effects were maintained at 3 and 6 month follow-up. We did not find significant post-test effects on fatigue or total sleep time.

**Conclusions:**

Findings indicate that guided online CBT-I is not only effective for insomnia complaints but also for depressive symptoms. The effects are large and comparable with those of depression therapy. Clinical trial registration number: NTR6049 (Netherlands Trial Register).

## Introduction

Insomnia and depression are both prevalent, severe disorders. Insomnia is characterized by difficulty initiating or maintaining sleep for three nights per week for 3 months, causing distress and impaired daily functioning (American Psychiatric Association, 2013). Depression is diagnosed when someone experiences depressed mood and/or loss of interest in daily activities for 2 weeks and shows four or more other specific symptoms (e.g. inactivity, concentration problems) along with impaired functioning (APA, 2013). Insomnia and depression are highly disturbing to a person's life and present significant financial burdens to society due to increased care consumption and decreased work productivity (Léger *et al.*, 2001; Daley *et al.*, 2009; Watkins *et al.*, 2009). Comorbidity is the rule rather than the exception; around 40% of people with insomnia suffer from clinical depression (Taylor *et al.*, 2005, 2007) and around 80% of depressed individuals suffer from insomnia symptoms (Ohayon, 2002; Szuba *et al.*, 2003; Franzen and Buysse, 2008).

Evidence is accumulating that insomnia plays a role in the development and maintenance of a depressive disorder. People with insomnia have a twofold chance of developing a depression (Baglioni *et al.*, 2011; Li *et al.*, 2018), insomnia perpetuates depressive disorders (Pigeon *et al.*, 2008), and residual insomnia complaints predict relapse after depression (Carney *et al.*, 2007). Suggested pathways through which insomnia affects depression include biological (e.g. sleep deprivation or fragmentation affecting the endocrine system), cognitive (e.g. effects of worrying and ruminating when lying awake), emotional (e.g. decreased emotional coping skills), and directly sleep-related pathways (e.g. use of sleep medication; Staner, 2010; Baglioni *et al.*, 2014; Finan *et al.*, 2015).

Despite the high comorbidity between insomnia and depression, it is still unclear which disorder should be treated (first) or whether combination treatments are more effective. What is clear is that an insomnia disorder is effectively treated with Cognitive Behavioral Therapy for Insomnia (CBT-I). Meta-analyses have demonstrated that the effects are rather small for total sleep time (TST; Hedges’ *g* = 0.16), but high for other sleep parameters such as sleep efficiency (SE; Hedges’ *g* = 0.71) and for insomnia severity (Hedges’ *g* = 0.98; Trauer *et al.*, 2015; Wu *et al.*, 2015; van Straten *et al.*, 2017). Long-term effects of CBT-I surpass the effects of sleep medication (Smith *et al.*, 2002; Riemann and Perlis, 2009). Because of growing demand for and shortage of trained therapists, as well as difficulties some patients encounter (e.g. travel time, disabilities) treatments are also offered online to enhance accessibility. Online CBT-I is administered effectively with effect sizes in the range of face-to-face treatment (Espie *et al.*, 2014; van Straten *et al.*, 2014; Zachariae *et al.*, 2016; Ritterband *et al.*, 2017), although direct comparisons between online and face-to-face CBT-I show mixed results (Blom *et al.*, 2015*b*; Lancee *et al.*, 2016). The effects of (online) CBT-I are not limited to sleep parameters and insomnia severity. Meta-analyses report moderate effects of insomnia treatment on comorbid depressive symptoms experienced by insomnia patients (*SMD* = 0.36 in Ye *et al.*, 2015; *d* = 0.34 in Ballesio *et al.*, 2017). However, most trials included in these meta-analyses did not target depressed populations specifically and in several studies patients with higher depression scores were excluded.

To our knowledge there have only been four studies that investigated CBT-I as a standalone treatment for people with both depressive symptoms and insomnia. Two of those studies used face-to-face treatment and both failed to demonstrate an effect of insomnia treatment on depressive symptoms compared with depression treatment-as-usual (Wagley *et al.*, 2013) and compared with relaxation therapy (Norell-Clarke *et al.*, 2015). The other two studies used online CBT-I as a standalone treatment for depressive symptoms and insomnia. Those studies showed more promising results. Blom and colleagues showed that online CBT-I was significantly more effective than online depression treatment on insomnia but as effective on depressive symptoms (Blom *et al.*, 2015*a*). However, the sample size of this study was small and replication is needed before conclusions can be drawn. Christensen and colleagues (2016) demonstrated that online CBT-I effectively reduced depressive symptoms and insomnia symptoms in patients with complaints of both compared with an online placebo module. However, people with higher scores of depressive symptoms were excluded (Patient Health Questionnaire-9 >20; Spitzer *et al.*, 1999).

We aimed to replicate the findings of Christensen and colleagues and expand them by not excluding people with higher levels of depressive symptoms, in a randomized controlled design comparing online CBT-I (i-Sleep) with a non-treated control group, monitoring their sleep daily. The online treatment included online feedback by a coach as this has been shown to potentiate efficacy (Lancee *et al.*, 2013*b*). We hypothesized that i-Sleep would be more effective than the control group in reducing depression symptoms. Secondarily, we investigated effects on insomnia severity, sleep diary parameters, daytime consequences of insomnia, fatigue, anxiety, and perseverative thinking. We hypothesized that i-Sleep would be more effective compared with the control group. We hypothesized that the effects of i-Sleep are generalized on functioning and other psychiatric symptoms such as perseverative thinking and anxiety known to be related to insomnia (Ohayon and Roth, 2003; Ehring and Watkins, 2008; Harvey *et al.*, 2017).

## Methods

### Participants and recruitment

**Fig. 1.**
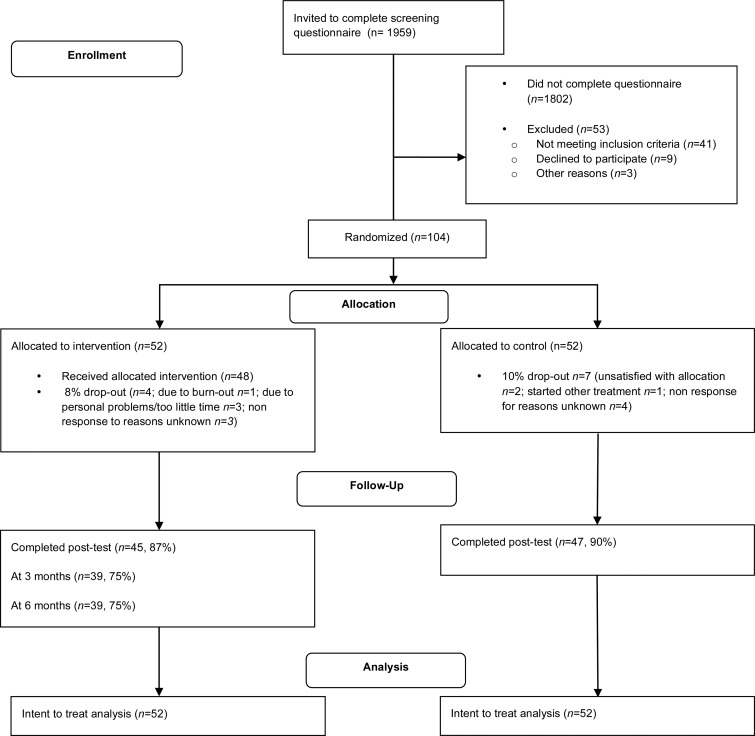
CONSORT 2010 Flow Diagram.

Participants who expressed interest in participating in research through a website on insomnia (www.insomnie.nl) were recruited. Potential participants received an email invitation to a screening questionnaire. Recruitment was completed in October 2016. The last follow-up occurred in June 2017 (see Fig. 1).

Inclusion criteria (assessed in the online screening) were: (1) ⩾18 years, (2) fulfilling DSM-5 criteria for insomnia (APA, 2013): trouble falling or staying asleep, ⩾ three nights a week, ⩾30 min, with significant consequences, sufficient opportunity to sleep and dissatisfaction with amount or quality of sleep; (3) depressive symptoms [PHQ-9 score >4; (Spitzer *et al.*, 1999)]; and (4) access to the Internet and email. Exclusion criteria were (1) probable sleep apnea (Wilson *et al.*, 2010); (2) previous CBT-I; (3) started psychotherapy < 6 months ago; (4) pregnancy/breastfeeding; (5) shift work; (6) being diagnosed with psychosis/schizophrenia; and (7) suicidal intentions [five items from the Mini International Neuropsychiatric Interview diagnostic interview (Sheehan *et al.*, 1998)]. Other psychiatric and somatic comorbidities were allowed, as were the use of sleep medication, melatonin, and homeopathic aids.

### Sample size

Based on the effect on depression (PHQ-9) of *d* = 0.69 reported by Christensen *et al.* (2016), a desired power of 0.8 and an alpha level of 0.05 (two-sided) a total of 34 participants per condition was needed. Considering risk of high dropout from online interventions (24% in Horsch *et al.*, 2015) we aimed to include 52 participants per condition.

### Procedure

After informed consent, participants completed the baseline questionnaire [demographics, Patient Health Questionnaire-9 (PHQ-9, Spitzer *et al.*, 1999), Insomnia Severity Index (ISI, Bastien *et al.*, 2001), consequences of insomnia during daytime (Espie *et al.*, 2012); Fatigue Severity Scale (FSS, Krupp *et al.*, 1989) and Hospital Anxiety and Depression Scale-Anxiety (HADS-A, Spinhoven *et al.*, 1997)]. Due to technical issues, the Perseverative Thinking Questionnaire (PTQ, Ehring *et al.*, 2012) scheduled at baseline was completed at week 1. Excluded participants were advised to contact their general practitioner. No applicants reported suicidal ideation. All eligible applicants were then asked to fill out a sleep diary. All participants that adhered to the diary (between 5 and 7 days) were included and subsequently randomized to (1) online CBT-I (i-Sleep) or (2) no treatment (sleep diary only). We used block randomization (blocks 2–4) with a 1:1 allocation sequence generated by an independent researcher also performing actual allocation. Blinding researchers or participants was not possible.

All participants were asked weekly to complete the PHQ-9 (Spitzer *et al.*, 1999), the ISI (Bastien *et al.* 2001), and the questionnaire on daily consequences (Espie *et al.*, 2012). In addition, we asked participants to complete a sleep diary every morning (Carney *et al.*, 2012).

Post-assessments occurred 9 weeks after randomization, for testing the primary hypothesis. Intervention participants received a 3 and 6-month follow-up assessment. The control group received treatment after post-assessment (week 9). Figure 1 shows a flow chart of the study. The study was registered at the Netherlands Trial Register (NTR6049). The University of Amsterdam Ethics Review Board approved the protocol (2016-CP-7263).

### Intervention

The online intervention i-Sleep consists of five sessions of CBT for insomnia (Morin and Espie, 2003; van der Zweerde *et al.*, 2016). The 5 sessions focused on (1) sleep hygiene and lifestyle, (2) stimulus control and sleep restriction therapy [SRT; in which the time in bed (TIB) is restricted to the average time slept in the last week (with a 5-h minimum), increasing TIB when efficiency is >85% and decreasing when it is <80%, with the aim of increasing the TIB spent asleep and decreasing TIB spent awake], (3) relaxation, (4) cognitive therapy tackling dysfunctional thoughts about sleep, (5) relapse prevention (van der Zweerde *et al.*, 2016). Clinical psychology graduate students at the University of Amsterdam offered online guidance (~40 min per participant per week) under weekly supervision by the first and last author. Online guidance entailed feedback on exercises, discussing SRT/bedtimes based on the diary and motivating participants to persevere in the treatment.

### Outcome measures

The primary outcome measure was the Dutch version of a nine-item depression scale, the Patient Health Questionnaire-9 at week 9 (PHQ-9; range 0–27, Cronbach's *α* = 0.94; Spitzer *et al.*, 1999). The PHQ-9 is scored on a five-point Likert score ranging from 0 (never) to 4 (almost daily). Next to the total score of the PHQ-9 we also report the total score without the sleep item, preventing measuring a decline in depression directly due to improved sleep (see also Christensen *et al.*, 2016). We call this the Patient Health Questionnaire-9 without sleep item (PHQ-WS) score.

Secondary measures were included to assess insomnia symptoms, daytime functioning and other psychological symptoms associated with insomnia and depression. The Insomnia Severity Index (ISI; Bastien *et al.*, 2001) is a seven item-scale scored on a five-point Likert scale. The total score ranges from 0 to 28. The ISI has good psychometric properties (Cronbach's *α* = 0.78). The daytime consequences of insomnia were measured with six items (energy, mood, concentration, sleepiness, productivity, and relationships). Each item was scored on five-point Likert scale (Espie *et al.*, 2012) and summed into one total score for daytime functioning. Fatigue was measured with the FSS (Cronbach's *α* = 0.89, nine statements scored on a seven-point Likert scale; Krupp *et al.*, 1989). Anxiety was measured with the Anxiety section of the Hospital Anxiety and Depression Scale (HADS-A; Cronbach's *α* = 0.84, seven items on a four-point Likert scale, scores ranging 0–21; Spinhoven *et al.*, 1997). Perseverative thinking was measured using the PTQ (Cronbach's *α* = 0.94; 15 items are rated on a four-point Likert scale, scores range 0–60; Ehring *et al.*, 2012). For all questionnaires, higher scores indicated more severe complaints.

### Sleep diary

The Carney consensus sleep diary (Carney *et al.*, 2012) was slightly adjusted to fit our study purpose and reduce participant burden. Participants reported when they went to bed to sleep and when they got up, sleep onset latency (SOL), TIB awake after sleep onset (WASO), how long they slept in total and mood on a scale of 1–10 (higher scores indicating better mood). We calculated TST (TST: TIB–SOL–WASO), and then calculated the SE (SE: TST/TIB × 100). Two items of the original Carney diary were left out: (1) the item distinguishing between the time patients went to bed and the time they switched off the light, and (2) the item distinguishing between final morning awakening and getting up.

### Adverse events

At follow-up, participants were asked about adverse events over the course of participation. They indicated whether they had fallen, had an (traffic) accident or experienced other negative events that seemed related to fatigue and/or sleepiness (and if yes, what happened and any physical or psychological consequences).

### Statistical methods

All randomized participants were included in the analyses following the intention-to-treat principle (Newell, 1992). Generalized mixed (multilevel) regression analysis was used to evaluate within-group effects (Time) and between-group effects (Time × Condition) of the intervention. Mixed negative binomial regression analyses with a log link were performed for skewed variables (PHQ-WS, ISI, PTQ, FSS, SE, SOL, WASO, TST), regular linear mixed regression with an identity link in all other cases. Two-level (participants and measurement points) repeated-measures designs were examined with outcomes as dependent variables (PHQ, PHQ-WS, ISI, daytime consequences, FSS, HADS-A, PTQ, sleep diary), Condition as between-subjects factor (intervention *v.* control) and Time as a within-subjects factor (pre- *v.* post-test). We used an unstructured covariance structure, which assumes data is missing at random, as the best model for the data and number of measurement points. Significance tests were performed to assess pre-treatment differences between groups. Baseline characteristics that significantly differed between groups and variables that predicted dropout were added as covariates. Analyses were repeated without covariates as a sensitivity analysis.

Within-group Cohen's *d* effect sizes were based on multilevel estimated baseline and post-test means (i.e., pre-post) and pooled observed standard deviations at baseline. Between-group Cohen's *d* effect sizes were calculated by dividing the difference in change scores (i.e., pre-post) by the pooled standard deviations at baseline (Morris, 2008). Cohen's *d* was considered small (i.e. <0.20), moderate (around 0.50) or large (⩾0.80; Cohen, 1988). Analyses were carried out using a 0.05 *α*-level (two-tailed) in SPSS v24.

## Results

### Descriptives and non-response

We included 104 patients (52 intervention and 52 control). The mean age of the sample was 45.99 years (s.d. = 12.32) and 82% of the participants were female (Table 1). Insomnia duration was around 10 years (M = 9.79, s.d. = 9.91). The majority of participants completed higher vocational or university education (60%). At baseline, participants showed mild (PHQ-9 > 4) to severe depression (PHQ-9 > 19) with scores ranging from 5 to 22 (M = 10.19, s.d. = 3.90; see online Supplementary Fig. S1). There were no baseline differences between groups with the exception of perseverative thinking (see online Supplementary Table S4). Those in the i-Sleep condition reported more baseline perseverative thinking (M = 32.35, s.d. = 1.53) than participants in the control condition (M = 28.69, s.d. = 1.73; *U* = 1568, *p* = 0.03). Age, depression scores (PHQ-9 and PHQ-WS), and TST proved to be associated with non-response at post-test in the treatment condition: younger patients, patients with higher depression scores and shorter TST were less likely to fill out the post-test. We included these variables as covariates in the regression models on all outcome measures. Non-response in the control condition was not related to any of the variables examined. At the follow-ups, baseline PTQ was associated with nonresponse (non-responders had more perseverative thinking) and was therefore included as a covariate in the analysis. Figure 1 shows a flowchart of the study.

**Table 1.** Demographics and pre-treatment characteristics

	i-Sleep (*n* = 52)	Sleep diary (*n* = 52)	
	M (s.e.)	M (s.e.)	Statistic
Age	44.64 (1.82)	46.29 (2.09)	*t*(102) = −0.691, *p* = 0.49
Insomnia duration (years)	9.92 (1.56)	9.10 (1.38)	*U* = *1023.5, p* = 0.76
Female sex, *n* (%)	43 (80.8%)	42 (82.7%)	χ^*2*^ = 0.064, *p* = 0.80
Antidepressant use, *n* (%)	8 (15.4%)	7 (13.5%)	χ^*2*^ = 0.078, *p* = 0.78
Prescription sleep med. use, *n* (%)	15 (28.8%)	15 (28.8%)	χ^*2*^ = 0.004, *p* = 0.95

### Treatment adherence, satisfaction, and adverse events

Most of the 52 intervention participants (*n* = 41, 85%) completed the full i-Sleep intervention. Four participants dropped out of the intervention (see flowchart, Fig. 1). The remaining participants completed 1 (*n* = 3), 2 (*n* = 1), or four sessions (*n* = 3).

On average patients were satisfied with the website (M = 7.67 on a scale 1–10, s.d. = 0.98), with the feedback (M = 7.91, s.d. = 0.87), and the online module (M = 7.91, s.d. = 0.82, see online Supplementary Table S1). No adverse events related to the intervention or trial were reported.

### Treatment effects on depression

**Fig. 2.**
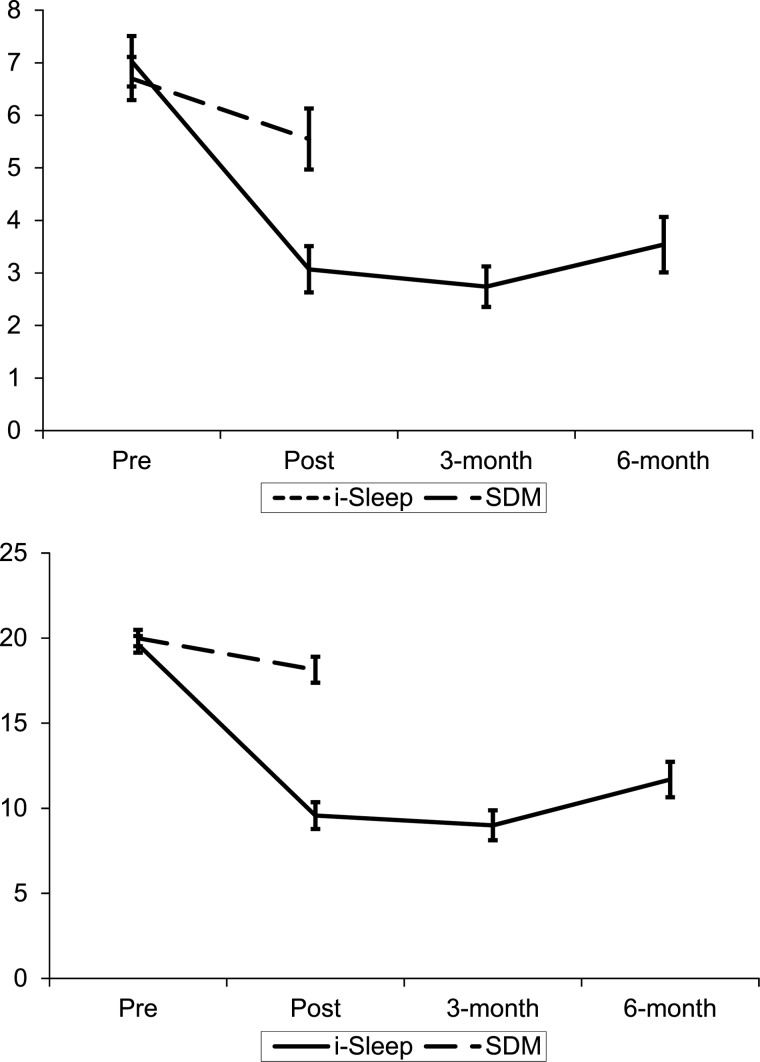
Mean PHQ-WS and ISI in both conditions at pre- and post-assessment and FU for i-Sleep condition. *Note*: Error bars represent 95% confidence intervals. PHQ-WS, Patient Health Questionnaire 9 minus the sleep item; ISI, Insomnia Severity Index; I-Sleep, online CBTI treatment; SDM, Sleep Diary Monitoring.

Multilevel regression analyses showed significant Time × Condition interaction effects for all three depression scores [PHQ-9, *F*_(1,81)_ = 20.54, *p* < 0.001 (Fig. 2); PHQ-WS, *F*_(1,82)_ = 14.38, *p* < 0.001, and the Mood item in the diary, *F*_(1,83)_ = 13.34, *p* < 0.001]. This indicates participants in the i-Sleep condition experienced a greater decline in depressive symptoms at post-test than the control condition (*d*_between_ = 1.05 for the total PHQ, *d*_between_ = 0.76 for the PHQ without the sleep item and *d*_between_ = 0.68 for the Mood item). Details of the analysis are in Table 2 and online Supplementary Tables S2–S5.

**Table 2.** (Mixed multilevel) regression-based pre, post, and FU estimated means and Cohen's *d* effect sizes*^a^

Measures	Pre, M (s.e.)	Post, M (s.e.)	FU 1 M (s.d.)	FU 2 M (s.d.)	Within Cohen's *d* pre *v.* post	Between Cohen's *d* pre *v.* post	Cohen's *d* pre *v.* FU 3 months	Cohen's *d* pre *v.* FU 6 months
*Questionnaires*								
PHQ-WS i-Sleep	7.03 (0.48)	3.07 (0.44)	2.86 (2.46)	3.61 (3.41)	−1.07	−0.76	−1.12	−0.92
PHQ-WS SDiary	6.70 (0.41)	5.55 (0.58)			−0.31			
PHQ-9 i-Sleep	9.94 (0.50)	4.41 (0.55)	4.00 (2.99)	5.00 (3.89)	−1.43	−1.05	−1.54	−1.28
PHQ-9 SDiary	9.35 (0.45)	7.88 (0.66)			−0.38			
ISI i-Sleep	19.63 (0.49)	9.57 (0.79)	9.17 (5.66)	12.03 (6.64)	−2.90	−2.36	−3.01	−2.19
ISI SDiary	20.00 (0.48)	18.14(0.76)			−0.54			
FSS i-Sleep	42.97 (1.26)	39.54 (1.37)	34.14 (10.63)	36.52 (10.23)	−0.36	−0.23	−0.92	−0.67
FSS SDiary	43.96 (1.21)	42.70 (1.33)			−0.13			
HADS i-Sleep	7.78 (0.30)	6.21 (0.50)	5.31 (2.77)	6.28 (4.12)	−0.52	−0.63	−0.81	−0.49
HADS SDiary	7.73 (0.36)	8.05 (0.54)			0.09			
DC i-Sleep	15.79 (0.34)	11.54 (0.59)	9.03 (4.27)	10.86 (3.67)	−1.40	−1.12	−2.23	−1.62
DC SDiary	15.08 (0.33)	14.22 (0.46)			−0.28			
PTQ i-Sleep	33.89 (1.40)	24.58 (1.87)	23.83 (12.48)	27.36 (14.60)	−0.81	−0.62	−0.87	−0.56
PTQ SDiary	30.81 (1.48)	28.63 (1.48)			−0.19			
*Diary variables*								
SE i-Sleep	68.18 (0.92)	82.44 (1.56)	82.60 (7.49)	79.00 (8.87)	1.19	0.67	1.20	0.90
SE SDiary	69.86 (0.94)	76.13 (1.58)			0.52			
TST i-Sleep	357.32 (8.30)	380.95 (7.87)	344.87 (66.82)	379.83 (45.42)	0.36	−0.05	−0.19	0.35
TST SDiary	339.79 (9.58)	359.96 (7.81)			0.31			
WASO i-Sleep	83.51 (5.86)	35.42 (3.58)	49.95 (32.50)	53.80 (34.73)	−0.91	−0.60	−0.63	−0.56
WASO SDiary	81.98 (5.54)	65.78 (5.75)			−0.31			
SOL i-Sleep	66.16 (6.40)	32.66 (4.93)	25.89 (15.72)	36.19 (29.17)	−0.74	−0.69	−0.89	−0.66
SOL SDiary	55.53 (5.85)	53.45 (6.90)			−0.05			
Mood i-Sleep	5.71 (0.13)	6.12 (0.17)	7.04 (0.62)	7.04 (0.54)	0.39	0.68	1.27	1.27
Mood SDiary	5.62 (0.12)	5.32 (0.16)			−0.29			

*A Cohen's *d* is commonly considered to be either small (i.e. <0.20), moderate (around 0.50) or large (0.80 and over; Cohen, 1988).

DC, daytime consequences; FU, follow-up; FSS, Fatigue Severity Scale; HADS-A, Hospital Anxiety and Depression Scale-anxiety subscale; I, i-Sleep treatment condition; ISI, Insomnia Severity Index; PHQ-9, Patient Health Questionnaire-9; PHQ-WS, Patient Health Questionnaire without Sleep item; PTQ, Perseverative Thinking Questionnaire; SE, Sleep Efficiency; SDiary, sleep diary condition; SOL, Sleep Onset Latency; TST, Total Sleep Time; WASO, Wake After Sleep Onset.

a Cohen's *d* using observed pooled standard deviation at baseline.

### Treatment effects on insomnia and fatigue

Significant Time × Condition interactions were also found for insomnia severity [ISI, *F*_(1,51)_ = 51.94, *p* < 0.001; Fig. 2] and for several sleep parameters from the diary [SE, *F*_(1,89)_ = 9.66, *p* < 0.001, SOL, *F*_(1,70)_ = 20.12, *p* < 0.001, and WASO, *F*_(1,80)_ = 22.59, *p* < 0.001]. This indicates that participants in the i-Sleep condition experienced more sleep improvements than participants in the control condition: they report lower insomnia severity (*d*_between_ = 2.36), higher SE (*d*_between_ = 0.67), lower SOL (*d*_between_ = 0.69) and less time spent WASO (*d*_between_ = 0.60). No significant Time × Condition effect could be found for TST, *F*_(1,73)_ = 0.04, *p* = 0.84, nor for Fatigue Severity, *F*_(1105)_ = 1.43, *p* = 0.24.

### Treatment effects on daytime functioning, anxiety, and perseverative thinking

Significant Time × Condition interactions were found on anxiety [HADS-A, *F*_(1,87)_ = 8.42, *p* < 0.01] perseverative thinking [PTQ, *F*_(1,91)_ = 9.48, *p* < 0.01] and daytime functioning, *F*_(1,88)_ = 18.44, *p* < 0.001. We observed no differential effects for the separate aspects of the daytime functioning and hence only report the total score. Findings indicate that participants in the i-Sleep condition reported less anxiety (*d*_between_ = 0.63), less perseverative thinking (*d*_between_ = 0.62) and better daytime functioning (*d*_between_ = 1.12) than participants in the control group.

### Treatment effects at follow-up

Non-significant time effects from post to follow-up assessments (using Greenhouse–Geisser correction where the sphericity assumption was violated) indicated that treatment effects at post assessment were largely maintained at follow-up assessment for depression [PHQ-9, *F*_(2,68)_ = 0.66, *p* = 0.52, PHQ-WS, *F*_(2,68)_ = 0.32, *p* = 0.73] and insomnia severity, *F*_(1.661, 56,48)_ = 0.29, *p* = 0.75. Effects on daytime functioning was maintained, *F*_(2,68)_ = 0.073, *p* = 0.93 as well as on comorbid psychological symptoms [anxiety, *F*_(2,68)_ = 0.29, *p* = 0.77, perseverative thinking, *F*_(1.626, 55.28)_ = 0.12, *p* = 0.94] and several sleep parameters [TST, *F*_(1.275,15.30)_ = 2.09, *p* = 0.15, WASO, *F*_(2,20)_ = 2.05, *p* = 0.16, SOL *F*_(2,22)_ = 2.49, *p* = 0.11 and SE, *F*_(2,18)_ = 2.60, *p* = 0.10]. There was no effect for fatigue at follow-up, *F*_(2,70)_ = 0.93, *p* = 0.4, while mood improved further, *F*_(2,24)_ = 5.73, *p* = 0.01, *d* = −0.23.

### Clinically relevant improvements

Participants in the i-Sleep condition showed significantly more clinically relevant improvements in both depression symptoms (clinical improvement defined as a drop of 50% or more resulting in <11; Spitzer *et al.*, 1999; 64% *v.* 30%) and insomnia severity (clinical improvement defined as a change of >8; Morin *et al.*, 2011; 64% *v.* 9%) than participants in the sleep diary monitoring control group. At 6 months follow-up, a considerable percentage of participants in the i-Sleep condition remained below the clinical cut-offs for depression (56%) and insomnia severity (50%) (see Table 3 for details).

**Table 3.** Clinical improvement on depressive symptoms and insomnia severity

	Baseline		Post-test			6 months follow-up
	i-Sleep (*n* = 52)	Sleep diary (*n* = 52)	i-Sleep (*n* = 45)	Sleep diary (*n* = 47)	Chi-square test of conditions (χ^2^)	i-Sleep (*n* = 39)
PHQ-9 > 10^a^	27 (52%)	21 (40%)	4 (9%)	13 (28%)	χ^2^ (1) = 5.38, *p* = 0.02	4 (10%)
PHQ-9 < 5^b^	0	0	28 (62%)	11 (23%)	χ^2^ (1) = 14.19, *p* < 0.001	22 (56%)
Clinically meaningful change PHQ-9^c^			29 (64%)	14 (30%)	χ^2^ (1) = 11.09, *p* < 0.001	29 (51%)
ISI > 10^d^	52 (100%)	52 (100%)	17 (38%)	43 (85%)	χ^2^ (1) = 29.24, *p* < 0.001	19 (50%)
Clinically meaningful change ISI^e^			29 (64%)	4 (9%)	χ^2^ (1) = 31.27, *p* < 0.001	29 (56%)

a Recommended cut-off point when using the PHQ-9 as a screener for depression (Kroenke and Spitzer, 2002).

b At least mild depressive symptoms, see inclusion criteria (Spitzer *et al.*, 1999).

c PHQ-9 drop of 50% or more resulting in a score below 10 (Spitzer *et al.*, 1999).

*Note*: at baseline, all participants had a PHQ-9 score of ⩾5 due to inclusion criteria.

d A clinical cut-off of 10 on the ISI is often used in insomnia research (Morin *et al.*, 2011).

e A change of 8 points or more; considered to be a clinically meaningful change (Morin *et al.*, 2011).

*Note*: At baseline, all participants had an ISI score of >10 due to the inclusion criteria.

## Discussion

Our primary aim was to investigate effects of online CBT-I on depressive symptoms in a sample of people with insomnia and at least a subclinical level of depression. We hypothesized that the online intervention i-Sleep would be more effective than no treatment (sleep diary monitoring only). Our findings convincingly show online CBT-I, reduces symptoms of depression and insomnia in people experiencing both.

Overall, the large effect sizes that we observed on depressive symptoms (PHQ-9, *d*_between_ = −1.05; PHQ-WS, *d*_between_ = −0.76) contrast with two face-to-face studies published in the past, which did not observe an effect on depression (Wagley *et al.*, 2013; Norell-Clarke *et al.*, 2015). The effects we found are larger than those observed in two meta-analyses on effects of (online) CBT-I for depressive symptoms (SMD = −0.36; Ye *et al.*, 2015*a*; *d* = 0.34 for individual CBT-I, *d* = 0.13 for self-help CBT-I; Ballesio *et al.*, 2017) and larger than in the Christensen study which also specifically examined the effects of CBT-I on depression. There could be several reasons why our observed effect on depressive symptoms was better than in other studies. First, many studies in the meta-analyses did not specifically target depression. Second, we had higher baseline depression severity scores in our sample than in the Christensen trial, which provides more room for improvement. Also, we used personalized feedback while the Christensen trial, and some other studies included in the meta-analyses, used automated feedback. Personalized feedback has been shown to enhance treatment effects (Lancee *et al.*, 2013*a*). Third and last, the Christensen trial used a more active control condition (Health Watch) than we did (monitoring only).

Our second aim was to explore effects of the treatment on insomnia. The observed effect on insomnia severity was comparable with a number of other online CBT-I trials but much larger (*d* = −2.36) than what was observed in the trial by Christensen and colleagues (*d* = −1.10, 2016) and in the most recent meta-analysis of (online) CBT (Hedges’ *g* = 1.03 in Zachariae *et al.*, 2016; Hedges’ *g* = 0.98 in van Straten *et al.*, 2017). Future research is needed to identify sources of this heterogeneity of treatment effect sizes. The effects on SE were moderate, but in line with observations in a recent meta-analysis (van Straten *et al.*, 2017). An increase in SE was also observed in the control condition (within Cohen's *d* = *0*.52, compared with *d* = 1.19 in the i-Sleep condition). It appears that merely keeping a sleep diary can have a positive effect on SE, although this result is tentative since we did not include a third group who did not fill out a sleep diary. This means that we cannot rule out that this increase in SE might also be caused by natural recovery.

Interestingly, results did not show that participants were less fatigued or slept more (TST) compared with participants in the control group. Perhaps, due to the SRT, participants were sleeping more efficiently and experiencing fewer symptoms (showing improvements on all symptom measures) at post-test, but were not recovered in such a way that they felt less tired. Possibly, the treatment causes participants to improve SE and therefore reduces insomnia complaints (i.e. decline on ISI, more satisfied with sleep, less trouble falling/staying asleep, etc.), while the SRT itself limited TST and actually caused fatigue. Fatigue is often an important reason for seeking treatment (Riedel and Lichstein, 2000) and is a commonly reported side effect of sleep restriction (Kyle *et al.*, 2011). However, if the fatigue could be interpreted as a side effect of treatment here, then we would have expected to see postponed improvements in fatigue after treatment, in line with other research (e.g. Vitiello *et al.*, 2014) we did not observe this after 6 months. Apparently insomnia severity and depression symptoms can largely improve despite fatigue remaining stable. Intuitively, it may seem that more and/or better sleep should automatically lead to more restoration of depleted resources and therefore less fatigue. But research has shown the relationship between fatigue and sleep to be more complex (Fortier-Brochu *et al.*, 2010). Future research may determine whether treatments targeting fatigue directly could be helpful in insomnia.

We found significant moderate to large improvements after online CBT-I treatment on our other outcomes intended to measure general (psychological) functioning: daytime functioning, anxiety, and perseverative thinking. It is interesting to note that although the treatment is not specifically aimed at these comorbid psychological symptoms, they do improve. The effect on daytime functioning (in energy, mood, concentration, sleepiness, productivity, and relationships) deserves specific attention because they are often overlooked in insomnia research (Kyle *et al.*, 2010, 2013). Suffering daytime consequences is one of the main reasons for seeking treatment (Morin *et al.*, 2006) and our findings suggest online CBT-I for people suffering from insomnia and depression has the potential to increase quality of life by improving daytime functioning. Future research will be necessary to confirm this (see e.g. protocol Espie *et al.*, 2016).

CBT-I treatment might be regarded as a treatment not purely targeting insomnia. Some of the components are clearly targeting sleep, for example stimulus-control and SRT. However, parts of the sleep hygiene component (i.e., including promoting physical exercise and a structured routine in the morning and at night) show overlap with behavioral activation, a treatment component of CBT for depression. Additionally, targeting dysfunctional thoughts about sleep resembles cognitive therapy for depression and may encourage patients to use this strategy on non-sleep related depressive thought patterns as well. Likewise, relaxation is also used in depression treatment (Beck, 1979). The question remains whether depression symptoms abate because sleep improves, or whether specific components of the treatment work towards improving depression symptoms directly. Which specific CBT-I components improve depressive complaints remains to be investigated. Dismantling studies could be used to isolate treatment ingredients that confer benefit to sleep and depressive symptoms, which may help refine treatment of the common comorbid presentation. A proportion of the participants in our study that underwent treatment remained above clinical cut-offs for depression (38%) and insomnia (38%) at post-test. Future studies should investigate how, possibly, combining protocols may enhance treatment response. Additionally, mediation and network approach research is needed to see whether sleep improves before, after or at the same time as depression does.

Effects were maintained at follow-up. At 6 months follow-up, 22 (56%) participants in the i-Sleep condition remained below the mild depression cut-off (PHQ-9 < 5), and 19 participants (50%) remained below the insomnia cut-off (⩽10) on the ISI. However, since we had a 25% non-response we cannot rule out that participants who did not complete the follow-up measurements were worse off than the ones that did (or neither that they were better off). Further research is needed to assess longer-term effects more reliably. Technical difficulties forced us to use a different program (Qualtrics, www.qualtrics.com) for the sleep diary at 6 months. This may have influenced the sleep diary adherence.

Some limitations of the present study need to be acknowledged. We actively recruited participants who were interested in insomnia treatment from the general population who were not clinically diagnosed for depression. The majority was female and highly educated. This is conceivably a very different population than patients that seek depression treatment through regular (mental) health care. However, this is also the case in other studies investigating the effect of online depression treatment on depressive symptoms that often use samples that show comparable baseline depression severity (e.g. Clarke *et al.*, 2009; Cavanagh *et al*., 2011; Titov *et al.*, 2015). The next step is to study whether online CBT-I treatment is also effective for patients with depressive symptoms in general (mental) health care settings, when patients turn to their general practitioner or psychologist for help. Also, in this study, we did not restrict time spent by clinical psychology students providing the feedback. Lancee *et al.* (2013*a*, 2013*b*) showed it is possible to complete guidance for the entire treatment in < 40 min. We expect trained professionals more experienced with patients will provide feedback in 15–20 min per session, lowering the necessary investment.

In summary, our study shows that guided online CBT-I can be used to treat people suffering from both insomnia and depression symptoms. In our sample with mild to moderate depressive symptoms, effects on depressive symptoms (PHQ-9, *d* = −1.05; PHQ-WS, *d* = *−*0.76) were larger than those found in a meta-analysis of supported online CBT for self-reported depressive symptoms (Andersson and Cuijpers, 2009). Our findings provide further evidence that insomnia should not be treated as a mere symptom of a depressive episode but requires dedicated attention. Targeting insomnia may offer new potential in the treatment of depression. Further research is crucial to clarify the specific relations between insomnia and depression symptoms, in patients with a clinical diagnosis of both, preferably including a depression treatment condition and using both self-report and objective measures such as actigraphy, to optimize therapeutic effects and improve clinical outcomes.

## Supplementary material

For supplementary material accompanying this paper visit https://doi.org/10.1017/S0033291718001149. click here to view supplementary material
