# Supplementary material for: Does online insomnia treatment reduce depressive symptoms? A randomized controlled trial in individuals with both insomnia and depressive symptoms
Source: Psychol Med. 2018 May 11;49(3):501–9. doi: 10.1017/S0033291718001149 (PMC6331685; doi:10.1017/S0033291718001149)
Supplement: Supplementary file 1 [file S0033291718001149sup001.pdf]

Supplementary Table S1. Evaluation of the treatment by participants in the i-Sleep condition (n=45).

	M (SD)
Overall grades (1-10)	
website	7.67 (.98)
feedback	7.91 (.87)
i-Sleep treatment	7.91 (.82)
Website characteristics (0-100%)	
clarity	75.69 (11.17)
speed	82.11 (15.88)
navigation	73.6 (14.21)
layout	78.3 (11.91)
Feedback evaluation (1-5)	
quality	4.29 (.55; lowest rating = 3)
length	4.31 (.47; lowest rating = 4)
frequency	4.13 (.55; lowest rating =3)
waiting time	4.12 (.69; lowest rating = 2)
i-Sleep treatment (1-5)	
explanations	4.27 (.50; lowest rating = 3)
examples	4.20 (.51; lowest rating = 3)
exercises	4.27 (.45; lowest rating = 4)
method	4.27 (.54; lowest rating = 3)
length	4.11 (.68; lowest rating = 2)
novelty	2.09 (.60; range 1-3)

Note. M = mean, SD = standard deviation. Overall grade is scored on a 1-10 scale, evaluation of feedback and treatment are scored on a 1-5 scale.

Supplementary Table S2. Multilevel regression models for all questionnaire measures

	PHQ-9	PHQ-S	ISI	DC	PTQ	HADS-A	FSS
F's and associated significance							
Model	25.04***	20.28***	79.66***	73.33***	75.09***	34.83***	26.32***
Time	61.50***	36.44***	89.82***	41.28***	23.93***	3.69	6.19*
Condition	5.01*	5.60*	33.38***	3.11	.13	2.75	1.36
Time × Condition	20.54***	14.38***	51.94***	18.44***	9.48**	8.42**	1.43
Age	19.63***	24.32***	.01	3.09	.41	4.57*	.019
PTQ	23.29***	29.04***	2.28	1.35	-	7.52*	1.96
PHQ-S	-	-	51.68***	46.97***	23.70***	32.14***	22.54***
TST	4.64*	3.59	14.23***	1.94	.86	.87	.08

Note.DC = daytime consequences; FSS = Fatigue Severity Scale; HADS-A = Hospital Anxiety and Depression Scale-anxiety subscale; I = i-Sleep treatment condition; ISI = Insomnia Severity Index; PHQ-9 = Patient Health Questionnaire-9; PHQ-S = Patient Health Questionnaire minus Sleep item; PTQ = Perseverative Thinking Questionnaire.
*p < .05; **p < .01; ***p <.001.

Supplementary Table S3. Multilevel regression models for all diary measures

	SE	SOL	WASO	TST	Mood
F's and associated significance					
Model	11.11***	28.35***	41.63***	2.74**	22.59***
Time	70.65***	25.08***	65.75***	15.81***	.35
Condition	2.53	1.06	8.53**	3.78	6.28*
Time * Condition	8.86**	20.12***	22.59***	.04	13.34**
Age	.10	1.99	4.52*	3.93*	3.79
PTQ	.03	.00	1.07	.00	1.04
PHQ-S	13.61***	15.45***	26.43***	4.90***	17.95***
TST	59.40***	8.55**	28.51***	-	3.41

Note. SE = Sleep Efficiency; SOL = Sleep Onset Latency; TST = Total Sleep Time; WASO = Wake After Sleep Onset. *p < .05; **p < .01; ***p < .001.

Supplementary Table S4. Baseline differences between drop-out vs. completers in both conditions

	i-Sleep (n=52, drop-out =7)		Control (n=52, drop-out =6)	
	mean (SE)	statistic	mean (SE)	statistic
Age		*t(50)*=2.527, *p*=.015*		*t(50)*=.705, *p*=.484
Dropout	35.14 (3.46)		43.33 (4.31)	
Completers	46.71 (1.72)		47.28 (1.94)	
Duration insomnia (cons.)		*t*(45)=-.742, *p*=.462		*t*(40)=-1.462, *p*=.152
Dropout	13.5 (5.74)		14.4 (4.35)	
Completers	9.90 (1.65)		8.41 (1.39)	
PHQ		*U*=70, *p*=.017*		*U*=117.5, *p*=.57
Dropout	14.29 (1.74)		10.33 (1.75)	
Completers	9.53 (.56)		9.43 (.51)	
PHQ-S		*U*=71.5, *p*=.019*		*U*=126.5, *p*=.748
Dropout	11.43 (1.70)		7.50 (1.69)	
Completers	6.98 (.54)		7.04 (.50)	
ISI[1]		*U*=131, *p*=.494		*U*=188, *p*=.160
Dropout	20.14 (1.58)		17.33 (1.09)	
Completers	18.96 (.55)		19.02 (.48)	
DC[2]		*U*=96, *p*=.103		*U*=139.5, *p*=.967
Dropout	16.71 (1.11)		13.50 (1.98)	
Completers	14.44 (.41)		13.89 (.44)	
HADS-A[3]		*U*=147.5, *p*=.793		*U*=87, *p*=.152
Dropout	7.71(1.32)		8.67 (1.09)	
Completers	7.44 (.47)		6.70 (.43)	
FSS[4]		*U*=116.5, *p*=.278		*U*=133.5, *p*=.900
Dropout	44.71(3.08)		41.33 (4.61)	
Completers	39.64 (1.49)		40.24 (1.38)	
PTQ[5]		*t*(50)=-.241, *p*=.811		*t*(46)=-.689, *p*=.494
Dropout	33.29 (1.68)		33.33 (9.06)	
Completers	32.20 (3.69)		28.38 (1.77)	
TST		*t*(50)=2.137, *p*=.038*		*t*(49)=-1.509, *p*=.138
Dropout	319.43 (32.03)		388.67 (39.25)	
Completers	365.13 (6.94)		340.53 (10.48)	

SE		*U*=214.5, *p*=.128		*U*=101.5, *p*=.327
Dropout	61.57 (6.72)		73.48 (6.35)	
Completers	71.49 (1.30)		69.24 (1.96)	
TIB		*t*(50)=-.531, *p*=.598		*t*(49)=-1.569, *p*=.123
Dropout	521.14 (15.18)		530.5 (19.46)	
Completers	511.58 (6.69)		492.64 (8.40)	
WASO		*U*=139.5, *p*=.636		*U*=158.5, *p*=.502
Dropout	114.43 (31.05)		85.96 (36.75)	
Completers	86.36 (6.08)		91.15 (8.08)	
Mood		*t*(50)=1.539, *p*=.13		*t*(49)=-.430, *p*=.669
Dropout	5.27 (.40)		6.15 (.45)	
Completers	5.94 (.16)		5.96 (.15)	

Note: DC = Daytime Consequences; FSS = Fatigue Severity Scale; HADS-A = Hospital Anxiety and Depression Scale-Anxiety subscale; I = i-Sleep treatment condition; ISI = Insomnia Severity Index; PHQ-9 = Patient Health Questionnaire-9; PHQ-S = Patient Health Questionnaire minus Sleep item; PTQ = Perseverative Thinking Questionnaire; SE = Sleep Efficiency; SOL = Sleep Onset Latency; TST = Total Sleep Time; WASO = Wake After Sleep Onset. *p<0.05, **p<.01. Standard errors are between parentheses. If assumptions for parametric tests were violated, non-parametric *Mann-Whitney* test was conducted. For dichotomous variables, Chi-square tests were carried out. [1] ISI 0-7 = no clinically significant insomnia, 8-14 = sub threshold insomnia, 15-21 = clinical insomnia (moderate severity), >21 = severe clinical insomnia. 2 Daytime Consequences scores range 0-24, higher scores indicating more severe consequences. [3]HADS-A subscale scores range from 0-21, normal = 0-7, mild 8-10, moderate 11-14 and severe 15-21. [4]FSS mean total score is reported. Scores >36 are commonly interpreted as reason to seek physician's evaluation. [5]PTQ scores range 0-60. Means differ for healthy student populations (M=28.63, SD=9.67) and depressed patients (M=37.56, SD=9.99) (Ehring et al., 2011).

Supplementary figure SF1. Frequencies of baseline PHQ-9 scores in the sample (n=104).

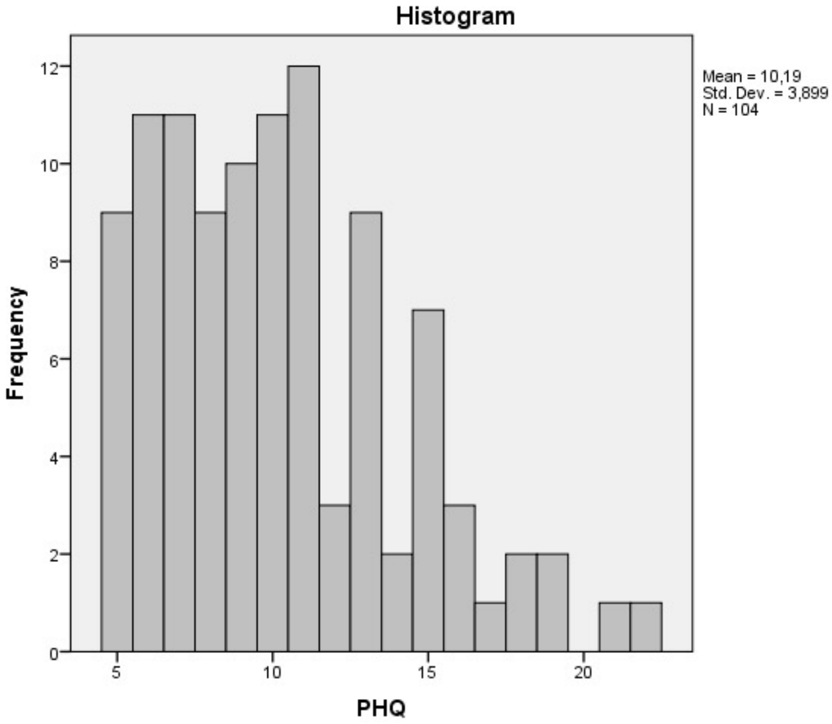

Supplementary Table S5. Observed means and *SD* (in parentheses) for all outcome measures

		Pre-assessment		Post-assessment		3-months		6-months	
Questionnaires		*n*	*M (SD)*	*n*	*M (SD)*	*n*	*M (SD)*	*n*	*M (SD)*
PHQ-9	I	52	10.1 (4.19)	45	4.20 (3.57)	39	3.85 (2.93)	39	4.9 (3.77)
	C	52	9.54 (3.53)	47	7.89 (4.67)				
PHQ-S	I	52	7.58 (3.99)	45	3.07 (3.09)	39	2.74 (2.40)	39	3.54 (3.30)
	C	52	7.10 (3.44)	47	5.74 (4.22)				
ISI	I	52	19.16 (3.76)	45	9.24 (5.41)	39	9.00 (5.50)	39	11.69 (6.50)
	C	52	18.83 (3.19)	47	17.09 (5.17)				
HADS-A	I	52	7.48 (3.16)	45	5.84 (4.10)	39	5.28 (2.70)	39	6.26 (4.15)
	C	52	6.92 (2.91)	47	7.11 (4.03)				
DC	I	52	14.75 (2.83)	45	10.20 (3.89)	39	8.90 (4.16)	39	10.72 (4.15)
	C	52	13.85 (3.19)	47	12.98 (3.71)				
PTQ	I	52	32.35 (11.00)	45	23.4 (12.88)	39	23.85 (12.08)	39	26.51 (14.48)
	C	48	28.69 (11.99)	47	26.60 (11.00)				
FSS	I	52	40.33 (9.83)	45	36.31 (10.89)	39	33.92 (10.23)	39	35.97 (10.79)
	C	52	40.37 (9.48)	47	39.06 (10.36)				
Sleep diary									
SE	I	52	70.15 (10.68)	42	85.57 (7.03)	19	84.50 (7.71)	25	78.72 (10.71)
	C	51	69.71 (13.35)	41	74.15 (11.05)				
SOL	I	52	64.04 (39.90)	42	28.14 (28.75)	18	25.08 (18.51)	28	46.77 (45.50)
	C	51	60.31 (50.50)	41	51.00 (42.34)				
WASO	I	52	90.13 (48.19)	42	38.05 (23.30)	17	43.72 (30.43)	27	49.24 (41.56)
	C	51	90.51 (58.25)	41	73.71 (47.45)				
TST	I	52	358.98 (54.45)	42	386.71 (47.97)	19	379.49 (54.91)	27	379.41 (53.89)
	C	51	346.20 (74.31)	41	355.73 (56.98)				
Mood	I	52	5.85 (1.09)	42	6.33 (1.28)	18	6.93 (.81)	26	6.66 (1.51)
	C	51	5.98 (1.02)	41	5.74 (1.11)				

Note. C = Control condition; DC = Daytime Consequences; FSS = Fatigue Severity Scale; HADS-A = Hospital Anxiety and Depression Scale-Anxiety subscale; I = i-Sleep treatment condition; ISI = Insomnia Severity Index; PHQ-9 = Patient Health Questionnaire-9; PHQ-S = Patient Health Questionnaire minus Sleep item; PTQ = Perseverative Thinking Questionnaire; SE = Sleep Efficiency; SOL = Sleep Onset Latency; TST = Total Sleep Time; WASO = Wake After Sleep Onset.
